# Supplementary material for: Prognostic association of immunoproteasome expression in solid tumours is governed by the immediate immune environment
Source: Mol Oncol. 2023 May 11;17(6):1041–59. doi: 10.1002/1878-0261.13443 (PMC10257426; doi:10.1002/1878-0261.13443)
Supplement: Supplementary file 1 — Fig. S1. Comparison of CP and IP expression in tumours with high purity (> 70%). Fig. S2. Single‐cell analysis of CP and IP expression across tumours. Fig. S3. Differential pathway enrichment between high‐ and low‐CP tumour samples. Fig. S4. Somatic copy number alterations in proteasome genes and their correlation with gene expression. Fig. S5. Association of CP expression with overall survival and response to immune checkpoint blockade (ICB) therapies. [file MOL2-17-1041-s009.pdf]

Supplementary Figures:

Supplementary Figure 1

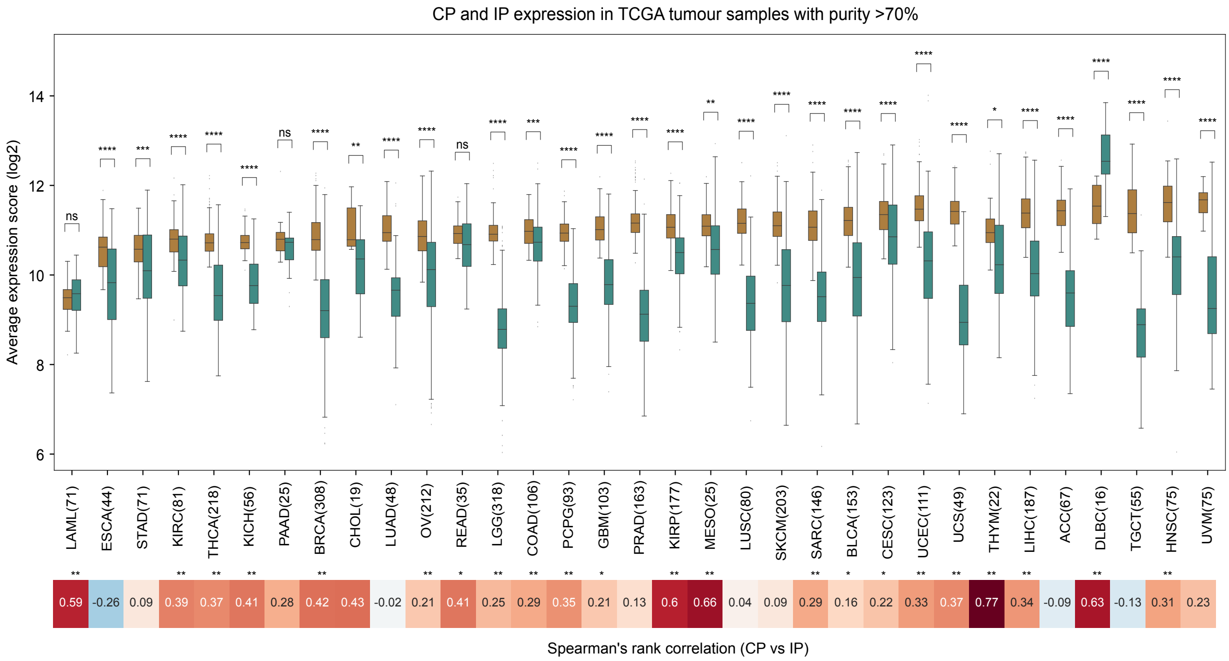

Supplementary Figure 1: Comparison of CP and IP expression in TCGA tumours with high purity (>70%)

The box plot shows the distribution of average expression of constitutive proteasome (CP) and immunoproteasome (IP) genes from 3,535 TCGA samples with high tumour-purity (>70%) across 33 different tumour tissues. The x-axis represents the tumour tissues (with the number of samples that have tumour-purity value >70%) and the y-axis represents the average expression level of CP (PSMB5, PSMB6 and PSMB7) and IP (PSMB8, PSMB9 and PSMB10) genes. The tumour-purity was calculated using the ABSOLUTE algorithm (see Methods). In each boxplot, the horizontal middle line indicates the median, the height of the shaded box indicates the interquartile range (IQR), and the whiskers indicate 1.5 x IQR. The P-value shown at the top, comparing IP and CP expression distributions at each tumour type, was computed using the Mann-Whitney U test (two-sided) and the significance level was represented as: **** P <= 0.0001, *** 0.0001 < P <= 0.001, ** 0.001 < P <= 0.01, * 0.01 < P <= 0.05 or ns - non-significant (P > 0.05). The heatmap at the bottom represents Spearman's rank correlation between average expression of CP and IP at the sample level for each tumour type. The tumour types that showed significant correlation were highlighted with the asterisks symbol on the top (** P <= 0.01 and * 0.01 < P <= 0.05).

Supplementary Figure 2

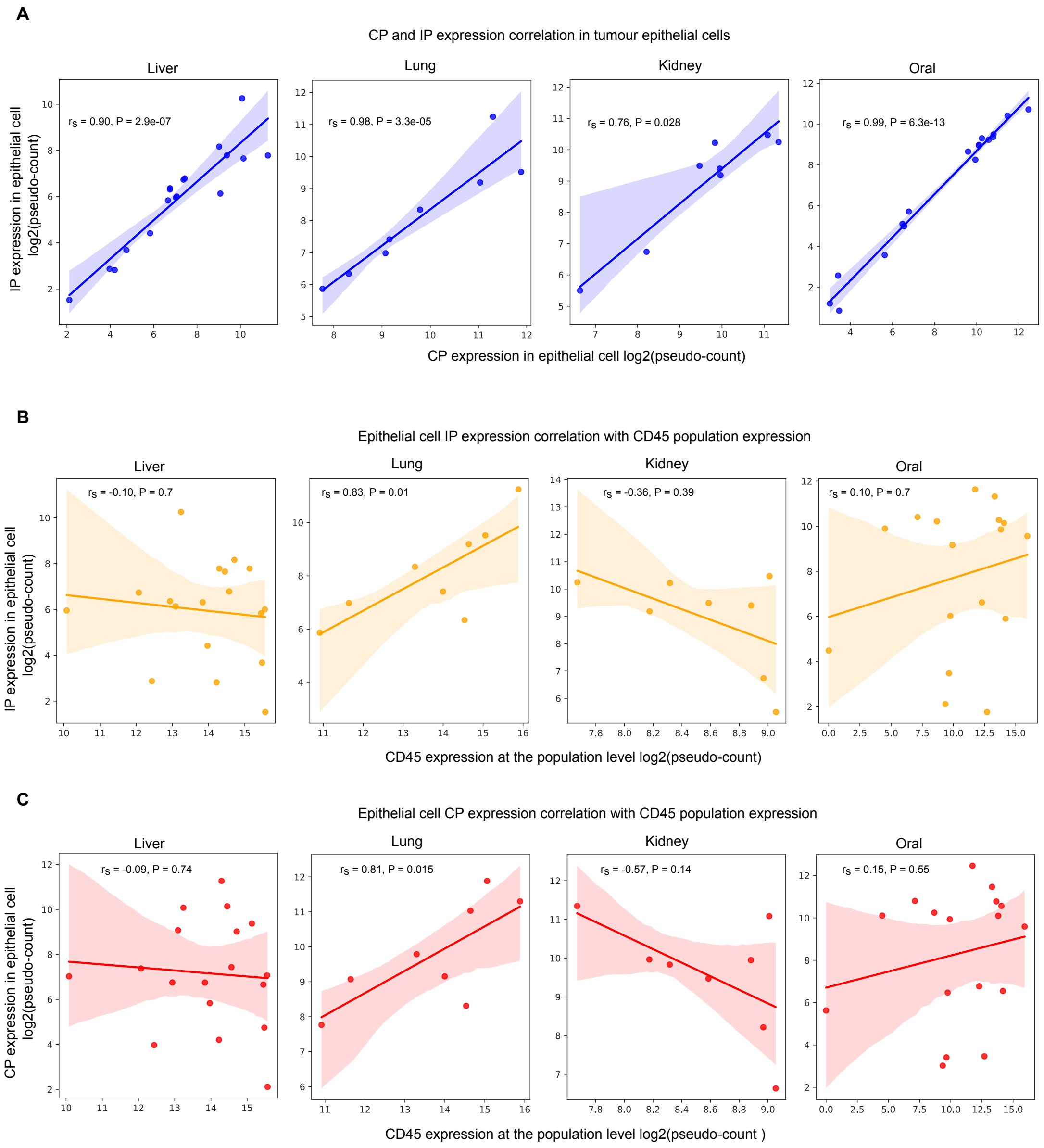

Supplementary Figure 2: Single-cell analysis of CP and IP expression across tumours

A. Spearman's rank correlation between the average expression of CP and IP genes (from tumour epithelial cell population) in four different cancer tissues (liver, lung, kidney and oral). Each dot on the plot represents a sample and the values on the x- and y-axis represent the CP and IP expression level, respectively, as pseud-count (i.e., average expression of CP or IP genes across all cells within the sample).

B. Spearman's rank correlation between CD45 gene expression (from all cells) and IP expression (from epithelial cells) at the sample level in four different cancer tissues (liver, lung, kidney and oral).

C. Same as B, but for CP.

Supplementary Figure 3

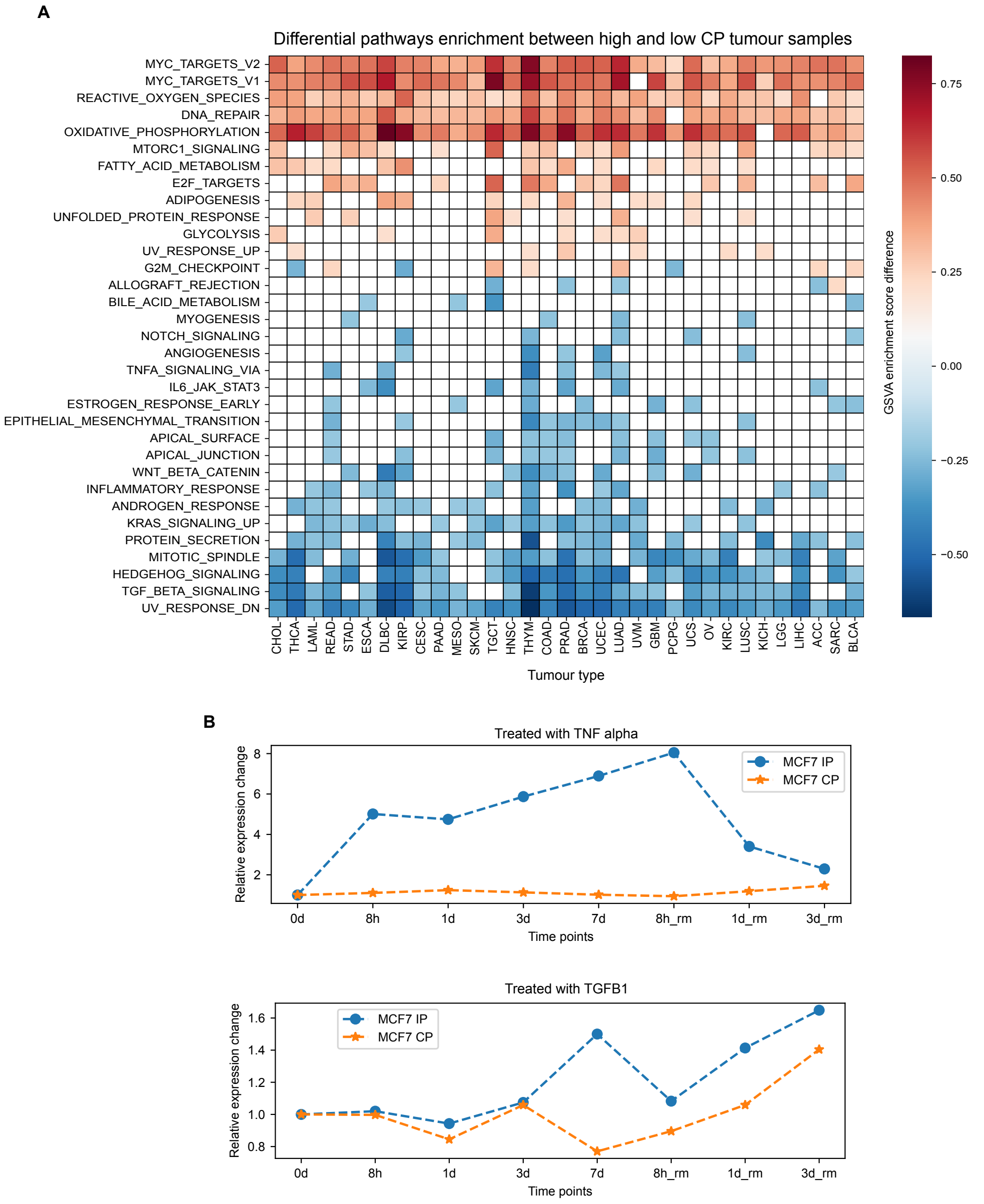

Supplementary Figure 3: Differential pathway enrichment between high and low CP tumour samples

A. Differentially enrichment of hallmark gene-set (from MsigDB) between samples of high-CP (upper quartile) and low-CP (lower quartile) expression groups. The x-axis represents the tumour type and the y-axis represents the different hallmark pathways. The colour of each cell in the heatmap represents the GSVA enrichment score difference between the high-CP and low-CP expression group. Only those pathway that have GSVA enrichment score difference of >|0.2| and FDR adjusted p-value < 0.05 were shown here. The cells with white colour indicate no significant enrichment of pathways in the corresponding tumour, with respect to the above condition.

B. Relative expression changes in the IP and CP expression level in MCF-7 breast cancer cell line. The x-axis represents the different time points: untreated (0), under treatment (8h, 1 day, 3 days, 7 days) and withdrawal (rm) of treatment after 7 days of treatment (8h, 1 day and 3 days). The y-axis represents the relative expression change with respect to untreated condition (0).

Supplementary Figure 4

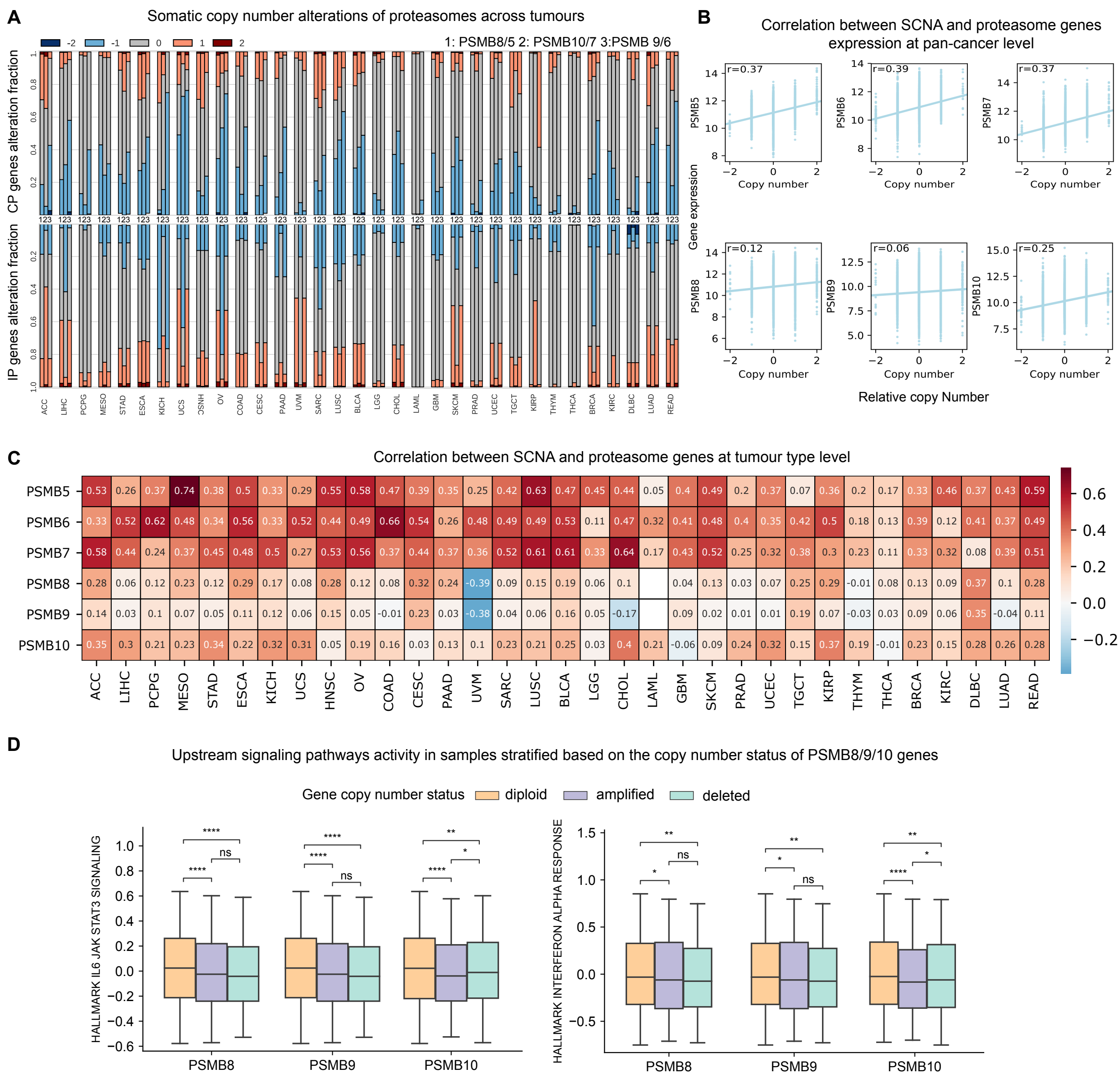

Supplementary Figure 4: Somatic copy number alterations in proteasome genes and their correlation with gene expression

A. Proportion of tumour samples having somatic copy number alteration in CP (PSMB5, PSMB6, PSMB7) and IP(PSMB8, PSMB9, PSMB10) genes. The stacked bar plot represents the fraction of samples with no somatic copy number alterations (zero), deletion (-1), deep deletion (-2), amplification (1) and high level amplification (2) in each tumour type. The top row is for CP genes and the columns 1, 2 and 3 represent PSMB5, PSMB7 and PSMB6, respectively. The bottom row is for IP genes and the columns 1, 2 and 3 represent PSMB8, PSMB10 and PSMB9, respectively.
B-C.Spearman's rank correlation between somatic copy number alteration and CP and IP genes individually at the pan-cancer level (**B**), and at the individual tumour type level shown in the bottom heatmap annotated with correlation values (**C**).
D. Expression level of IL6_JAK_STAT3 and Interferon alpha response pathways (computed using GSVA) in samples with IP genes copy number altered (amplified CNA>0 or deleted CNA<0) and unaltered (CNA==0, diploid). The p-values were computed using the Mann-Whitney U test (two-sided) and the significance level was represented as: **** P <= 0.0001, *** 0.0001 < P <= 0.001, ** 0.001 < P <= 0.01, * 0.01 < P <= 0.05 or ns - non-significant (P > 0.05).

Supplementary Figure 5

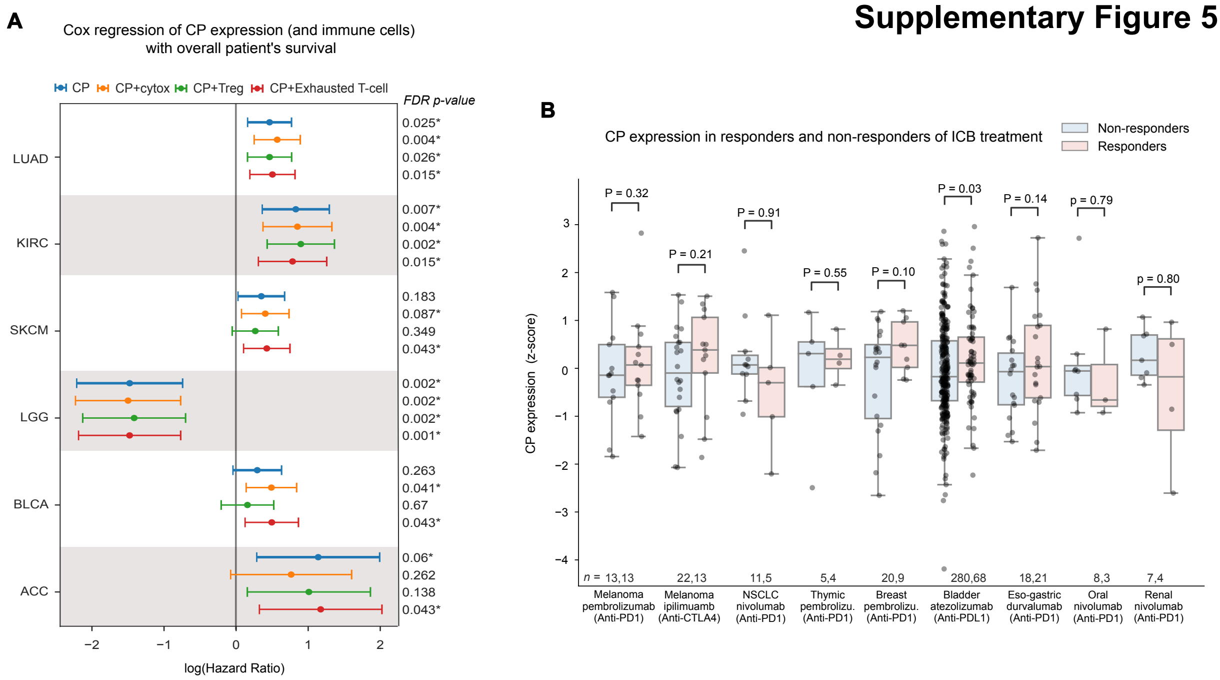

Supplementary Figure 5: Association of CP expression with overall survival and response to immune checkpoint blockade (ICB) therapies

A. Cox proportional hazard ratio (HR) for CP expression alone and in combination with three other confounders (regulatory T cells, Cytotoxic cells and exhausted T cells) in six different tumours from TCGA which showed significant prognostic association. The x-axis represents the log(HR) value (> 0 indicates bad prognosis and < 0 indicates good prognosis), and the horizontal line represents the mean and 95% confidence interval. The colour of the line indicates the variables considered for the analysis. The FDR adjusted p-value shown on the right was calculated using the Wald test and corrected for multiple hypothesis testing using Benjamini-Hochberg method. The asterisks(*) symbol indicates FDR adjusted p-value less than 10% significance level.

B. The boxplot shows the average CP expression (represented as z-scores) in responders and non-responders of ICB therapies in eight different tumour types. One-sided Mann-Whitney U test was used to compare whether the CP expression in responder was higher than non-responder, and the corresponding P-values were mentioned at the top.
